# Analysis of Nonlinear Gene Expression Progression Reveals Extensive Pathway and Age-Specific Transitions in Aging Human Brains

**DOI:** 10.1371/journal.pone.0074578

**Published:** 2013-10-03

**Authors:** Kajia Cao, Paul Ryvkin, Yih-Chii Hwang, F. Brad Johnson, Li-San Wang

**Affiliations:** 1 Department of Pathology and Laboratory Medicine, University of Pennsylvania, Philadelphia, Pennsylvania, United States of America; 2 Genomics and Computational Biology Graduate Group, University of Pennsylvania, Philadelphia, Pennsylvania, United States of America; 3 Penn Center for Bioinformatics, University of Pennsylvania, Philadelphia, Pennsylvania, United States of America; 4 Institute on Aging, University of Pennsylvania, Philadelphia, Pennsylvania, United States of America; Tokyo Medical and Dental University, Japan

## Abstract

Several recent gene expression studies identified hundreds of genes that are correlated with age in brain and other tissues in human. However, these studies used linear models of age correlation, which are not well equipped to model abrupt changes associated with particular ages. We developed a computational algorithm for age estimation in which the expression of each gene is treated as a dichotomized biomarker for whether the subject is older or younger than a particular age. In addition, for each age-informative gene our algorithm identifies the age threshold with the most drastic change in expression level, which allows us to associate genes with particular age periods. Analysis of human aging brain expression datasets from three frontal cortex regions showed that different pathways undergo transitions at different ages, and the distribution of pathways and age thresholds varies across brain regions. Our study reveals age-correlated expression changes at particular age points and allows one to estimate the age of an individual with better accuracy than previously published methods.

## Introduction

Aging is a multi-faceted, highly complex dynamic phenomenon that has influences on human biology and medicine. An important research problem in the study of aging is the identification of biomarkers that reflect aging-related physiological changes that can be further applied to the estimation of physiological age in an individual. With the advent of technology allowing for genome-wide surveys of gene expression, studies of aging biomarkers have been able to move beyond the use of classical physiological variables. Some examples include forced vital capacity (amount of air exhaled from the lungs after the deepest breath possible), systolic blood pressure, red blood cell count, hemoglobin, glucose, and blood urea nitrogen [1]–[3]. By correlating gene expression levels with age, hundreds of genes associated with the aging process have been identified in nematode [4], mosquito [5], fly [6]–[9], yeast [10], mouse [11], and human brain, muscle, and kidney [12]–[14]. The expression level of these genes can be used as age biomarkers to model the physiological age of organisms [5], [15], detect tissue-specific aging differences [12], [13], [16], [17] and study the biology of aging-related diseases [18], [19].

Among all the quantitative models used in these aging studies, the linear model is most widely used [4], [13], [14], [20]; for example, linear regression has been applied to estimate age-correlated genes in human kidney, muscle and brain [13], [14], [16], [21]. In our previous study on human brain aging [21], we developed an “age ruler” by applying regression models to gene expression in normal brain tissues. Application of the age ruler to brain tissues with neurodegeneration showed that Frontotemporal lobar dementia (FTLD) and Alzheimer's disease (AD) patients have gene expression profiles similar to those of healthy individuals with chronological ages older than the actual ages of the patients; i.e the FTLD and AD patients displayed signs of premature aging. Similarly, a piecewise linear regression model has been used in such aging studies in nematode and human brain tissues [4], [16], [22]. Golden et al. identified *C. elegans* genes correlated with age-related behavioral phenotypes using linear models and demonstrated that these are biomarkers of physiological age [4]. Their result showed difference between estimated physiological age and chronological age to be approximately five days. Sibille et al. identified age-related transcriptional changes in two human prefrontal cortex regions (Brodmann Area (BA) 9 and 47), and showed the residual in the regression prediction, i.e. the difference between the estimated physiological age and chronological age, was between 10 to 15 years.

A critical drawback of linear models is that, although they are useful in estimating the performance of a gene across the entire age range, they are not sensitive enough to detect nonlinear, abrupt expression changes at particular chronological ages. In addition, gene expression levels or other age-related traits such as genotype frequencies may not be linearly correlated with age or even monotonically ordered by age. This problem can be further exacerbated in population studies. For example, Bergman *et al.* observed that the percentage of deleterious genotypes in the population follows a “U-shape trend”: the percentage goes down as the population ages, but bounces back for the very old population (>90 y), potentially due to some unobserved “buffer mechanisms” that contribute to the longevity of these individuals through compensating for the deleterious effect [23]. There is clearly a need for new approaches to identify genes undergoing nonlinear transitions at particular age points.

### 1.1 Outline of the paper

In this study, we developed a novel computational approach that identifies genes from a reference dataset that undergo relatively abrupt transitions in expression at different ages, and which combines information from these genes to estimate the age of an individual using a naïve Bayes (NB) model. The algorithm decides the number of age-correlated genes for each age threshold by “greedily” adding the most informative genes until a pre-specified number of genes is reached, and then incorporates these genes into a NB classifier for age estimation. In addition, we tested other machine learning methods including linear regression (standard lasso or stepwise) and polynomial spline regression (POLYMARS) on the same data sets and demonstrated that our method outperformed these models. The estimation of chronological age using our method was up to 34% more accurate than the other tested methods. In addition, we assigned each gene to a decade from the range of 20 to 79 years based on the chronological age at which its expression undergoes the biggest change, in order to differentiate between genes whose expression profiles transition at different ages. Pathway and protein-protein interaction network analysis identified many pathway-specific changes associated with different stages of the aging process.

## Materials and Methods

### 2.1 Data preparation

Microarray datasets used in this paper were downloaded from GEO (http://www.ncbi.nlm.nih.gov/geo/index.cgi), or obtained from the authors directly. Table 1 summarizes the data used in this paper. For all datasets, the GCRMA package [24] for R/Bioconductor [25] was used to generate log-2 expression levels for probeset IDs from the original .cel files.

**Table 1:** Microarray datasets used in this study.

ID	tissue	Number of samples	Age range	Gender	GEO ID
H1	Rostral aspect of frontal cortex	29	26∼95	F: 11	GDS707 [12]
	(∼BA10)			M: 18	
H2	Dorsolateral prefrontal cortex	29	25∼79	F: 7	* [16]
	(BA9)			M: 22	
H3	Orbital prefrontal cortex	27	28∼77	F: 6	* [16]
	(BA47)			M: 21	

* : obtained from the authors directly.

### 2.2 A naïve Bayes approach predicts age by dichotomizing age

We developed a new computational approach for gene expression-based chronological age estimation by treating each gene as a binary classifier for whether an individual is older than a particular age threshold, and combining all such age-informative genes using a Naïve Bayes (NB) model [26], [27]. An NB model assumes all features are independently distributed, and combines their distributions to calculate the posterior probability of the outcome using the Bayes theorem. Naïve Bayes models are known to be robust even when the independence assumption is violated, and are widely used in high-dimensional applications, particularly studies involving global gene expression [28], [29].

Our algorithm works as follows. Given a dataset, the algorithm first sorts the ages of individuals from young to old, and determine “age thresholds” as the average ages for all pairs of adjacent ages. For each age threshold, the algorithm uses Fisher's exact test to identify genes having age-related expression levels that can differentiate between individuals older or younger than the threshold. Each of these genes is thus a binary classifier for that particular age threshold. Information from all selected age-informative genes is then aggregated using Bayes formula as a strong classifier to estimate age.

Consider a dataset in which there are no ties in age. The algorithm first sorts individuals of age *a_1_, a_2_, … a_n_* in ascending order, then defined Δ = {*δ_j_*: *j = 1,2,…,n*}as the set of *age thresholds*, in which 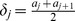, where 1≤*j*≤*n-1*. For each age threshold, the algorithm uses Fisher's exact test to identify genes that can differentiate between individuals older than the threshold from younger ones by expression level. To obtain binary classifiers for all age thresholds, we need to perform Fisher's exact test on each gene and each age threshold. The algorithm first computes the contingency table (Table 2) for probeset *g_ij_* at age *a = δ_j_* where *j≤m* samples (*m = 1, 2, …n-1*).

**Table 2:** Contingency table for probeset *g_ij_* at age *a = δ_j_* where *j≤m* samples (*m = 1, 2, …n-1*).

	*a_j_≤δ_j′_*	*a_j_>δ_j′_*
*g_ij_ ≤θ_i_*	*C_00_*	*C_01_*
*g_ij_>θ_i_*	*C_10_*	*C_11_*

Here, *θ_i_ = g_i1_, g_i2_,…g_im_* as a set of gene expression thresholds for each gene *i* across *m* samples selected by exhaust search; *a_j_* is the chronological age for individual *j*. This contingency table summarizes the number of genes have been counted under the corresponding conditions. The algorithm then computes the odds ratio
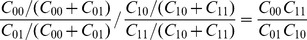
(1)and Fisher's exact test to get the significance of association between age threshold *δ_j_* and probeset *i* in sample *j*.

Using Bayes theorem, we write our model as follows:
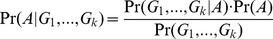
(2)In our study, we estimate the age threshold *a* with maximum 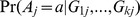 or individual *j* using:
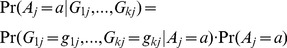
(3)


Here, gene *g_ij_* is the log-2 gene expression level of probe set *i* in individual *j*, age threshold *a ∈ δ* , *k* is the number of features (genes) obtained using Fisher's exact test.

Assuming individuals are uniformly distributed on age, we rewrite the formula
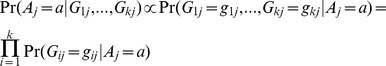
(4)Here, 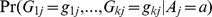 measures the association between gene expression of each gene and every age threshold in *δ*. 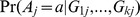 can be computed as follows:
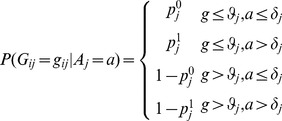
(5)


We use a greedy algorithm to select the features (genes) for each age threshold in age estimation to guarantee each selected gene was designated to at most one age threshold. The purpose of this selection step is to satisfy the requirement of the Bayes theorem (i.e., each gene is used at most once for a particular age threshold) while ensuring that every age threshold has enough genes assigned. The algorithm is as follows:

1. Consider table A where column represents age threshold and rows represent all the genes. Entry *o_ij_* in table A is the absolute value of log_2_ odds ratio for gene i and age threshold *j*.
2. Construct table B where each column corresponds to an age threshold and each row represents a gene that may be selected for each age threshold. The number of rows is *N*. Entry *b_ij_* in table B indicates gene *j* is assigned as the classifier for the *i^th^* age threshold. Initially every entry in B is set to 0.
3. Iterate over all columns. For each column *j* in table A, find the gene with the largest odds ratio (say gene *m*). Set *b_ij_* = 1. Change *o_mj_* = −Inf so that gene *m* will not be chosen for any other age threshold. After the iteration, each column in B will have one more entry set to 1.
4. Repeat step 3 until every column in B has *N* entries set to 1.

The source code in R is posted on our lab website (http://wanglab.pcbi.upenn.edu/nonlinear-aging/).

### 2.3 Age estimators using linear model

For performance comparison, we also used other statistical methods including linear regression, the lasso algorithm, and forward stepwise regression to compute the significance of a correlation between age and the expression level of a gene, with/without adjusting for the effect of gender. These approaches assume a linear relationship between age and log-2 expression level with additional features to control for model complexity and avoid overfitting. We compute lasso and stepwise regression using the R/lars package [30]. Specifically, we compute linear regression while adjusting for the effect of gender using the following equation: 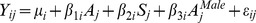
(6)Here *Y_ij_* is the log-2 gene expression level of probe set *i* in sample *j*, *A_j_* is the age for individual *j*, *S_j_* is 0 if individual *j* is female, 1 if he is male. *A_j_^Male^* is the age of individual *j* if *S_j_* = 1; it is 0 otherwise (included to test for interaction between age and gender). The coefficients *β_1i_*, *β_2i_*, and *β_3i_* are regression coefficients reflecting the rate of change in gene expression with respect to age alone, gender alone, and age-gender interaction effects, respectively.

The lasso is a shrinkage and selection method for linear regression. R package “lars” implements modified least angle regression and forward stepwise regression as the solutions for lasso [30]. We used the R package “lars” to compute the correlation between age and the expression level of a gene by forward stepwise regression and lasso. We also ran five-fold cross validation to estimate the age estimation errors. In each fold of cross validation, we first estimated the best tuning parameter such that the mean squared error is minimal by another five-fold cross validation on the training data, and then we estimated age in the testing data using the selected tuning parameter.

POLYMARS is the multivariate adaptive polynomial spline regression using piecewise linear splines to model the response [31]. It has been used to estimate age in a *C. elegans* study [4]. We included this method in our study to compare its performance with our naïve Bayes model.

### 2.4 Performance of age estimators by permutation test

We used five-fold cross validation to estimate the error of our age estimator as follows. We first divided the subjects into five subsets. We then used reference data from four of the subsets (training set) to generate age estimations for the individuals in the fifth subset (test set). Thus, the average estimation error over all five subsets is the estimated error of the age estimator:
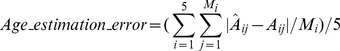
(7)Here *M_i_* is the total number of individuals in the *i^th^* partition (test set), and *A_ij_* and 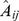 are the actual age and the age estimation for the *j^th^* individual in the *i^th^* partition respectively. We computed the significance of the error by obtaining 1,000 randomized cross-validation errors with age information randomly shuffled; the significance of the estimation error is the proportion of the 1,000 errors from the randomized dataset lower than the actual cross-validation error.

### 2.5 Dividing age-correlated genes into young, middle-age and old gene groups by their age transition points

Our NB algorithm is able to find the age threshold that has the biggest transition for each age-correlated gene, defined as the *transition age point* for the gene. We ran our NB classifier on the three brain data sets (H1∼H3) (Table 1) and obtained genes demonstrating significant statistical differences at each age threshold in each data set with p<0.005. For each of the three brain regions we divided age-correlated genes into three age groups: young (<40 years old), middle-age (between 40 and 59 years old) and old (between 60 and 79 years old).

### 2.6 The distribution of age correlated genes in the protein-protein interaction network

We generated a human whole protein-protein interaction (PPI) network, *G* = (*V*, *E*) using the largest connected component from Human Protein Reference Database (HPRD) Release 9 [32]. The PPI network *G* contains [2] = 9,267 proteins with [2] = 36,913 interactions. For each age-correlated gene corresponding protein in the network, we measured how “central” each node is within the network using four commonly used node statistics:

1. Degree (*k*) is defined as
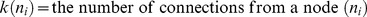
(8)
2. Betweenness Centrality (*B*) is defined as the ratio of shortest paths that go through a node (n_i_)
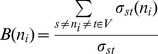
(9)where *σ_st_* = number of shortest paths from node *s* to node *t, δ_st_*(*n_i_*) = number of shortest paths from node *s* to node *t* through node *n_i_*.
3. Closeness Centrality (*Clo*) is defined as the average distance from the particular node to all the other nodes in the whole network:
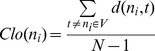
(10)where *N* is the number of nodes in a network, and *d*(*n_i_*, *t*) = the shortest path from node *n_i_* to all the other nodes in the network
4. Clustering Coefficient (*Clu*) is defined as the proportion of neighbor's actual connections among the number of neighbor's fully connections:
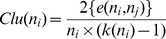
(11)where *e*(*n_i_*, *n_j_*) = edge from node *n_i_* to node *n_j_*.

For each set of genes, we define the *characteristic path length* as the average length of all pairwise shortest paths between every pair of genes in the set.
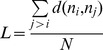
(12)where *N* is the number of nodes in a age-group and *d*(*n_i_*, *n_j_*) is the shortest path length of gene *n_i_* and *n_j_*.

Similar to what we did within a group, we amended the equation to measure the characteristic path length between two age groups
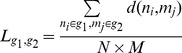
(13)where *g_1_* and *g_2_* denote the two groups we are looking at, and *N_g1_* is the number of nodes in an age-group *g_1_* and *M_g1_* is the number of nodes in the other age-group *g_2_*, and *d*(*n_i_*, *m_j_*) is the shortest path length of gene *n_i_* and *m_j_*.

Intuitively, the larger the statistic is, the more distant the genes in the set are from each other. Similarly, we define the characteristic path length between two sets of genes as the average length of all pairwise shortest paths between every pair of genes, one each from the two sets, and use this statistic to measure how proximate the two gene sets are on the network. We used these statistics to measure how close the three gene groups by age transition are similar to one another. We tested the statistical significance of each statistic using a permutation test by comparing the observed score with that computed using 1000 randomly generated gene sets of the same size. The same permutation test was performed for the characteristic path length statistics using 100 randomly generated gene sets/pairs of gene sets, as computing the characteristic path lengths took considerably more time.

### 2.7 Functional annotation analysis

The functional annotation analysis was done by submitting the gene lists to NIH DAVID online tool (http://david.abcc.ncifcrf.gov/summary.jsp). The statistical significances of functional annotations were calculated by Fishers' exact test. To reduce redundancy, we also used Functional Annotation Clustering in DAVID to group similar annotations together. The grouping algorithm is based on the hypothesis that similar annotations should have similar gene members. The enrichment score of each cluster that was used to rank its biological significance is the geometric mean (in −log scale) of member's p-values in a corresponding annotation cluster. In our analysis, we applied enrichment score cut-off at 3 (equivalent to p-value<0.001) to select the clusters of annotations.

## Results

### 3.1 Age estimation using the naïve Bayes approach on human brain gene expression

We tested our NB age estimation model using the three normal human brain datasets (H1∼H3) (Table 1), each consisting of gene expression profiles from three different areas of the frontal cortex: the rostral cortex (Dataset H1 for the Brodman Area 10 (BA10) region, see Methods section) [12], the dorsolateral prefrontal cortex (H2 for the BA9 brain region), and the orbital prefrontal cortex (H3, BA47) [16]. The ages of the subjects in each dataset were uniformly distributed between 25 and 95 years old. Accuracy in age estimation was evaluated using five-fold cross validation. We compared the errors of age estimation using four settings selected by our algorithm (*N* = 10, 20, 50 and 70) for the number of genes correlated to each age threshold in our estimator. The best *N* is the one with the least error in the five-fold cross validation. We found that optimal *N* varies across all three regions (Table 3), although N has very little impact on the estimation accuracy for BA9 and BA47. The best age estimation in BA10 was accurate to within 14.43 years of the actual age of the subject with *N* = 70; age estimation could be accurate to within 9.48 years in BA9 with *N* = 50; and age estimation error was 8.33 years in BA47 using *N* = 10 (Table 3). Among all three data sets, age estimation error in BA10 (H1) is largest (14.38 years). One possible explanation is that the number of probesets on the microarray platform (Affymetrix hgu95av2) used for this dataset is only half that of the platform (Affymetrix hgu133a) used for H2 and H3.

**Table 3:** Best number of genes (*N*) used in age estimation and the difference of median of age is the absolute difference between the median of estimated age and the median of chronological age.

	BA10	BA9	BA47
Error in age estimation (five-fold cross validation)	14.43±11.13	9.48±6.85	8.33±7.56
Best *N*	70	50	10
Difference of median of age (with actual age)	9.5	0.5	0.5
p values (permutation test)	0.009	0.002	0.001

The significance of the error was determined by obtaining 1,000 randomized cross-validation errors with age information randomly shuffled; the significance of the prediction error is the fraction of the 1,000 randomized errors lower than the actual cross-validation error. The best *N* varies across regions.

To determine the statistical significance of our predictions, we randomly permuted the ages of the individuals in each brain dataset (1,000 permutations performed) and examined the estimated errors: less than 0.9% of the permutations had lower age estimation errors than our observed error (8.33∼14.43 years) (Table 3). Therefore, our age estimators at each age threshold performed significantly better than by random chance. We also found that the difference between actual and estimated median age on the same group of subjects in the cross validation is very small (0.5 years) in BA9 (H2) and BA47 (H3), but relatively large (9.5 years) in BA10 (H1), probably due to a smaller sample size and fewer probesets on the microarray platform.

### 3.2 The Naïve Bayes approach outperforms other models for age estimation

**Figure 1:**
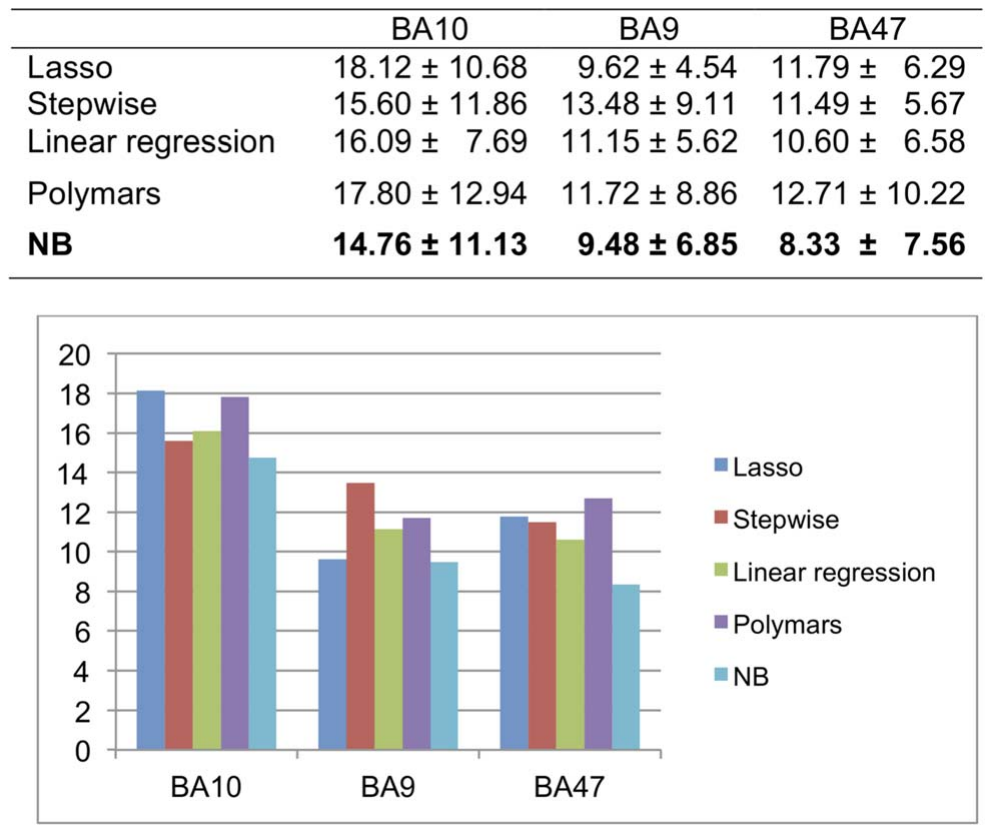
Performance of age estimation using the proposed naïve Bayes method and other methods. The five-fold cross validation results showed age estimation errors of the naïve Bayes model to be 0.14∼4.38 years smaller than in other tested models, thus reducing error by 34%.

Having demonstrated the accuracy of age estimation using our NB model, we next compared its performance with other widely used statistical models including linear regression (standard, lasso, and stepwise) [13], [14], and the POLYMARS (multivariate adaptive polynomial spline regression) method [4], on datasets H1∼H3. Our five-fold cross validation results showed age estimation errors of the NB model to be 0.14∼4.38 years less than other tested models, or up to 34.5% reduction in error (Figure 1).

We also tested our NB model on a nematode time course data set (GSE12290) published in [2]. In the paper, the authors applied POLYMARS to identify the gene correlated with age in c.elegans and showed difference between estimated physiological age and chronological age to be approximately five years. We applied NB model and other two models (linear regression and POLYMARS) to the data set. By testing five-fold cross validation, we found the estimated age using NB model is 3.06±2.03 years (N = 20) outperformed 4.46±3.34 years using linear regression and 3.56±2.68 years using POLYMARS. We also estimated the age estimation error using larger number of N, we found estimated age is 3.07±1.99 years when N = 50 and 3.01±1.96 years when N = 70. Therefore, the optimal N is determined as 70 genes in the three validation tests (N = 20, 50, and 70).

### 3.3 Genes down-regulated with age undergoing significant transition at middle age

**Figure 2:**
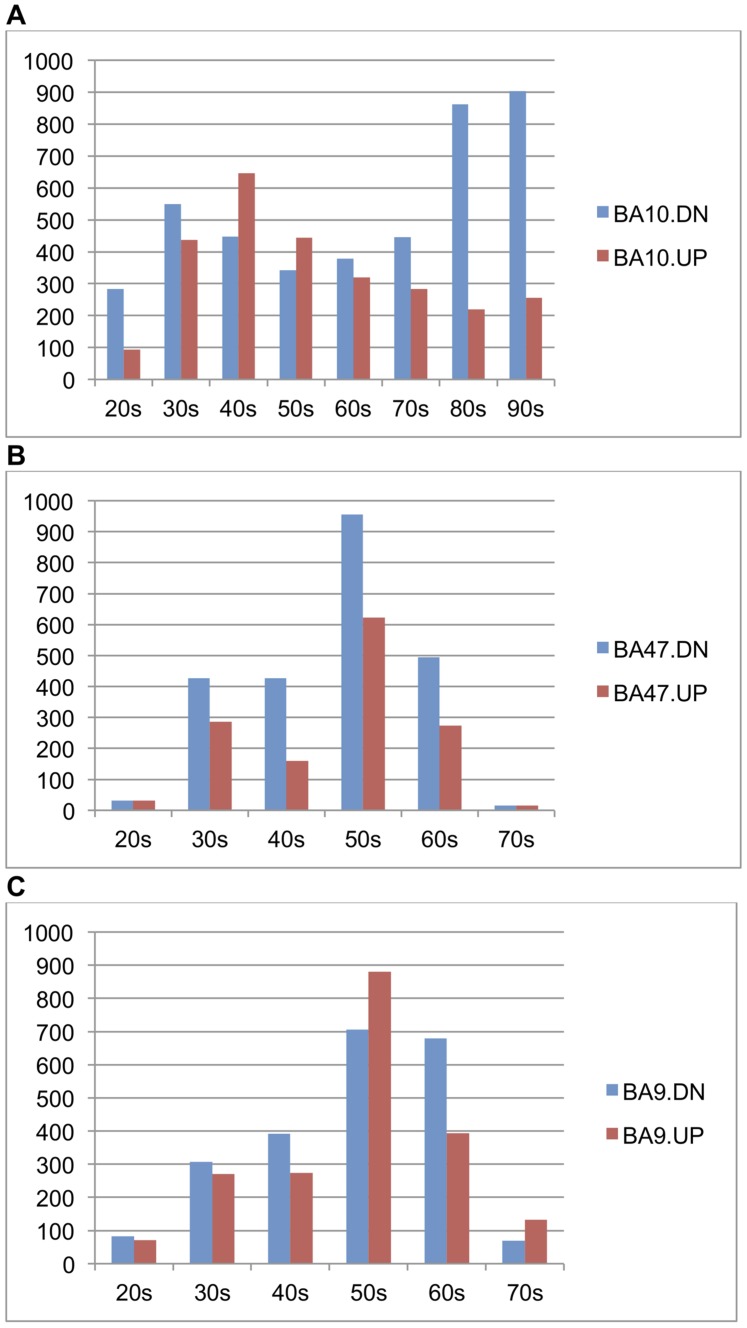
Histograms of genes with age-regulated transition points within each decade between 25 and 95 years (p-value≤0.005) in three brain regions. The distribution of age-regulated genes is very different in BA10 compared to BA9 and BA47.

A feature of our NB approach is that each age-correlated gene is associated with an age threshold with the biggest change in expression level. With this property, we are able to identify the *transition age point*, defined as the age thresholds at which a gene undergoes the most drastic expression change. We ran our NB classifier on the three brain data sets (H1∼H3) and obtained genes demonstrating significant statistical differences at each age threshold in each data set with p<0.005; the number of significant genes were 2190 for H1 (false discovery rate (FDR)≤0.026), 2458 for H2 (FDR≤0.041), and 2379 for H3 (FDR≤0.043). We analyzed the distributions of genes and their transition age points by the following steps. First, we merged the gene sets corresponding to transition age points that fall into the same decade and obtained the list of genes associated for each decade. Second, we generated histograms to show the distribution of these gene lists on each decade for three data sets. We compared the histograms of the same age range for the three brain regions (20 s∼70 s) (Figure 2). The histograms showed these age-correlated genes are not uniformly distributed by age for all three data sets. Specifically, we found that most genes undergoing transition are down-regulated with age in three regions and that the most common transition was during the 50 s for BA9 (H2) and BA47 (H3) but a decade earlier in BA10 (H1).

For each of the three brain regions we merged age-correlated genes into three age groups: young (<40 years old), middle-age (between 40 and 59 years old) and old (between 60 and 79 years old). We examined the biological significance of the three groups of age-correlated genes in each of the three brain regions using Gene Ontology [33] and KEGG (Kyoto Encyclopedia of Genes and Genomes) Pathway [34]–[36] annotation analysis by the NIAID DAVID database [37]. We selected annotation terms with significant transitions in at least two brain regions. These terms can be clustered into five major categories (total 13 categories, Table 4) based on their direction of change, the age by which the most drastic change occurs, and brain region: *synaptic function*, *fatty acid/membrane metabolism*, *mitochondria, purine nucleotide binding* and *actin cytoskeleton*. Globally, we found that the majority of the significant pathways show down regulation with age. Furthermore, with few exceptions, a pathway significant in two brain regions will have the same direction of change in both regions, although the age transition point may differ. Analysis results are generally in agreement with literature evidence: age-regulated reduction in neuronal genes that are involved in synaptic function are found to contribute to age-related cognitive change [38]–[40]. Fatty acid pathways are known to play a role in limiting cognitive decline during aging [41]–[44], and the observation of down-regulation is consistent with observations that certain cognitive abilities decline as a person ages [44]. Some studies have demonstrated that mitochondrial RNA levels are decreased in human brain aging [45] and oxidants generated by mitochondria appear to be the major source of the oxidative lesions that accumulate with age [46], [47]. The category “actin cytoskeleton” shows the connection of suggesting a connection between regulation of autophagy, axon guidance and actin dynamics [48] and plays a role in the development and maintenance of neuronal function in brain aging [49]. All these published observations are consistent with our findings. A full list of significant KEGG and GO terms are listed in Table S1.

**Table 4:** Functional annotation analysis summary.

			BA10						BA47						BA9					
Cluster	Description	No. pathways	Y		M		O		Y		M		O		Y		M		O	
			Dn	Up	Dn	Up	Dn	Up	Dn	Up	Dn	Up	Dn	Up	Dn	Up	Dn	Up	Dn	Up
**1**	**Synapse**	**40**	*		*						*				*		*		*	
2	Fatty acid/membrane metabolism	4				*														
**3**	**Mitochondria**	**24**					*		*				*							
**4**	**Purine nucleotide binding**	**24**	*								*				*		*		*	
5	Actin cytoskeleton	**4**		*																
**6**	**Ubiquitin proteolysis; channel activity**	**49**									*						*		*	*
**7**	Translation; DNA damage response	**5**										*						*		
8	Transcriptional activation	**12**												*	*		*			
9	Neuronal function	**49**															*		*	
10	Mitochondrial transport	**9**																	*	
11	Cytoskeleton/RNA splicing	**8**																		*
12	Calcium transport	**4**													*					
13	Chromatin regulation	**4**														*				

In the above analysis, we focused on gene changes at age thresholds that are younger than 80 years in BA10 (H1) in order to cross compare with the other two data sets where all individuals are younger than 80 years old (Figure 2). We carried out the same analysis for genes associated with 80 s and 90 s (“very old”) in BA10, and identified genes differentially expressed at every decade from 20 to 90 for BA10 for comparison (bars of 80 s and 90 s in Figure 2A). Interestingly, in BA10 the genes associated with the very old group showed substantially more genes down-regulated with age. More than 70% of the down-regulated genes in “very old” group were also found in genes down-regulated with age in the young group. We also found that more than 63% of genes up-regulated with age in the very old group overlapped with genes going in the same direction in the young group. Functional annotation analysis using DAVID showed that the young group and very old group in BA10 both tend to be involved in nucleotide or, more specifically, purine nucleotide (ATP/GTP) binding, but this was not true in the middle-age and old groups.

### 3.4 Associations between age and gene expression levels show similarity across brain regions in human frontal cortex, though functional difference exists

**Figure 3:**
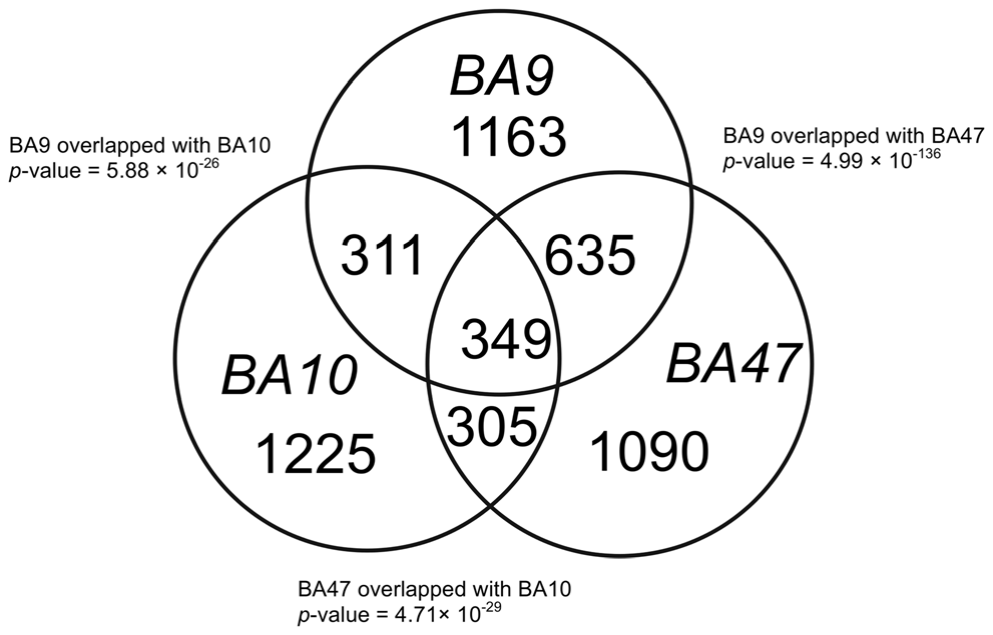
Venn diagram of three human brain regions. Age-correlated genes (p≤0.005) in three human brain regions have statistically significant overlaps. Shown are the numbers of genes with age-correlated expression in each brain region.

We combined all age-correlated genes in the young, middle-age and old groups in each data set and examined the overlaps among the three datasets. We found that out of more than 2000 genes with age-regulated gene expression in each Brodmann Area, 984 genes showed age-correlated gene expression in both BA9 and BA47, 660 genes showed age-correlated gene expression in both BA9 and BA10, and BA47 and BA10 had 654 genes in common (Figure 3). Fisher's exact test showed that all the pair-wise overlaps were statistically significant (*P*-value<6×10^−26^). Although the Venn diagram showed overlaps between BA10 and the other two regions are statistically significant, our annotation analysis using DAVID showed that BA10 is functionally different from the other two brain regions, while BA9 and BA47 have more similar biological functions (protein localization and transport).

### 3.5 Distribution characteristics of age correlated genes in the protein-protein interaction network

Having examined the functional significance and overlap of age-correlated genes and the effect of transition age points on these properties, we next analyzed the distribution of these genes on the protein-protein interaction (PPI) network to characterize how much these genes interact. Protein-protein interaction gives the binding relationship between proteins and indicates shared biological activities and functions. To systematically understand the functional relationships among age-correlated genes (*P*-value<0.005) for every decade in age by our NB approach, we first mapped the age-correlated genes to the Human Protein Reference Database (HPRD) PPI network [32], and analyzed four commonly used topological properties: degree, betweenness, closeness, and clustering coefficient. These properties are defined to measure where the proteins are located and how central or critical they are in the PPI network [50]–[52].

We used the same age-correlated gene grouping as in the pathway analysis for the network analysis. Out of 5,073 age-correlated genes, 3497 were found in the PPI network. We first calculated the four node centrality properties of all 9267 nodes in the PPI network including degree (mean = 7.97), betweenness centrality (mean = 3.49×10^−4^), closeness centrality (mean = 0.24), and clustering coefficient (mean = 0.11). We extracted the centrality properties of nine lists of age-correlated genes (combination of the three age transition time points and three brain regions). Permutation tests showed most groups have significantly higher degree (*z*-score>3), betweenness (*z*-score>3), and closeness centrality (*z*-score>3) when compared to random gene lists of the same size (Table 5). This result suggests that age-correlated genes are in central positions in the PPI network, and the high numbers of connections indicate these genes tend to interact with more genes than average (i.e. they have a high degree). These genes are involved in more biological pathways (have high betweenness centrality), and are also close to each other on the network (have high closeness centrality).

**Table 5:** Topological Characteristics of the protein-protein interaction network of age-correlated genes.

	Topological Characteristics in Protein-Protein Interaction Network											
	Degree			Betweenness Centrality			Closeness Centrality			Clustering Coefficient		
	Young	Middle-aged	Old	Young	Middle-aged	Old	Young	Middle-aged	Old	Young	Middle-aged	Old
BA10	13.083	11.894	12.645	6.74E-04	6.14E-04	7.05E-04	0.250	0.249	0.250	0.097	0.103	0.100
Z-score	**10.412**	**8.761**	**9.009**	**6.787**	**5.874**	**6.968**	**9.863**	**8.798**	**8.827**	−1.212	−0.322	−0.750
BA9	10.237	10.774	10.844	5.15E-04	5.45E-04	5.30E-04	0.247	0.247	0.250	0.094	0.101	0.094
*Z*-score	**3.665**	**7.087**	**5.806**	2.669	**5.103**	**3.720**	**4.804**	**8.124**	**8.546**	−1.318	−0.726	−1.595
BA47	11.254	10.516	8.573	6.29E-04	5.29E-04	3.78E-04	0.247	0.246	0.244	0.102	0.102	0.087
*Z* -score	**5.635**	**6.495**	0.940	**4.868**	**4.593**	0.453	**5.130**	**7.212**	2.705	−0.406	−0.612	−2.074

For each of the three brain regions age-correlated genes divided into three age groups: young (<40 years old), middle-age (between 40 and 59 years old) and old (between 60 and 79 years old).

We further divided each group into up-regulated and down-regulated gene sets using the sign of the log2-transformed odds-ratio obtained earlier when building the NB model (Table S2). The new gene lists showed even higher central roles (higher *z*-score) in three properties than the combining both the up- and down-regulated groups. Again, none of the gene groups showed statistical significance in clustering coefficient (absolute *z*-score<3). No notable tendency has been found for the genes when participating in any potential protein modules or complexes.

In order to inspect any biological function clustering within and between different age-correlated gene groups, we examined how closely the nodes are connected to each other by calculating the average characteristic path length for age-correlated genes in the PPI network. As shown in Table 6, almost all gene pairs in each brain region have path lengths (between 3.895 and 4.082), which is shorter than “the shortest path length” (4.24) of the network. This indicates that these age-correlated genes are more closely connected with each other than other interacted genes in the network. We next computed the characteristic path lengths between-groups (Method Section) of the three age-correlated gene groups (young, middle-age, and old) and obtained *z*-scores (<−5) by permuting the age-correlated genes in the network. We found that *z*-scores of the characteristic path lengths are significantly lower in all three groups and between every two groups. This is expected because of the substantial overlap among the lists of age-correlated genes in the three cortex regions.

**Table 6:** Characteristics Path Length in-between Groups of age-correlated genes.

		BA10			BA9			BA47		
		size	mean	*Z*-score	size	mean	*Z*-score	size	mean	*Z*-score
Whole Region Overlapped		1644	3.948	−10.043	1703	4.003	−8.834	1640	4.039	−6.937
Group of age correlated genes	**Y**oung	833	3.895	−9.014	482	3.978	−5.208	567	4.008	−4.344
	**M**id	1000	3.938	−8.145	1189	3.993	−6.941	1197	4.018	−6.998
	**O**ld	770	3.897	−7.929	831	3.909	−8.828	513	4.082	−2.776
	Y vs. M	1358	3.919	−11.666	1413	3.990	−7.604	1384	4.017	−6.858
	M vs. O	1336	3.920	−12.679	1501	3.953	−11.465	1463	4.056	−6.000
	O vs. Y	1300	3.901	−11.170	1186	3.950	−9.251	1013	4.055	−5.198

The size of each region shows the number of genes having overlap between the protein-protein interaction network and our age-regulated gene study. Size of Y(young) vs. M (middle-aged), M vs. O (old), and O vs. Y showed the union gene number of each group each brain region.

## Discussion

The proposed naïve Bayes model is our attempt to develop a method for more accurate age estimation. Our method is also an extension of our previously published age estimation model [21]. In that paper, we reported our aging study on the same three brain regions using linear regression. With the same *p*-value cut-off at 0.005, our NB method showed more sensitivity than linear regression and the numbers of genes shared between regions were found to be significantly greater; furthermore the NB method allowed us to discern a greater number of significant functional associations.

We observed diversity of age transition point and brain region specificity in the three datasets. These specificities are significantly associated with pathways (e.g. Table 4) for down-regulated genes; although there are as many up-regulated genes, the number of significant pathways is much smaller. This suggests a considerable portion of age-correlated gene expression changes reflects more than just random breakdown. These findings affirm the rich complexity in human brain aging, the value of these high-quality gene expression datasets, and the importance of reanalyzing these preexisting datasets using newer algorithms.

It is worth noting that the choice of *N*, the number of features in the NB model, can affect the performance of age estimation. In our study we used a standard “model selection” approach in machine learning literature: we determined the best of four different settings of *N* features (probe sets) for each age threshold by choosing the one with the best performance on the training data. It is important to note that having more features does not necessarily mean the accuracy will improve because over-fitting can occur.

An underlying assumption of our approach is that global gene expression reflects the true “physiologic age” of an individual. Given that independent physiological age biomarkers are not available for these datasets, chronological ages are used as an informative proxy of physiologic age in our cross validation verification. Because some individuals appear to age more rapidly than others, for any particular individual there may not be a perfect correspondence between the individual's predicted physiological and actual chronological age. However, for a population of individuals, there should be a good correspondence between these values. This is also in agreement with the finding that the error in predicting population average is much lower than error for individuals.

We merged age-correlated genes by combining decades into young, middle-age and old age groups, and found that BA9 shared biological functions with BA47 in the middle-age group and with BA10 in young group. We also found that BA47 shared genes involved in phosphorylation processes in the young and old groups while both the young and old groups in BA10 are involved in neuron projection and synaptic transmission (Table S1). Synaptic transmission has been shown to be partly unaffected or even compensatory in the aging process in the rodent hippocampus [53].

Notably, our analysis demonstrated that genes transitioning in the young group shared functional roles with genes in the very old group in BA10, including neuron projection, and ATP binding. The presence of genes involved in the morphogenesis of neuron projections may imply a change in the aging brain's ability to form connections between neurons, or that this process plays a protective role. The finding of age-correlated genes being involved in ATP binding is consistent with many studies showing that the efficiency of these processes is modulated by both aging [54] and AD [55], and it is also consistent with the role of oxidative stress in neuronal cell death [56]. It is also possible that these similarities between the gene sets associated with young and very old indicate survivor phenotypes, i.e. a lack of youthful gene expression in middle age may be linked to an early demise.

Our analysis showed that the associations between age and gene expression levels are substantially shared among the three human frontal cortex regions (Figure 2), yet there were apparent functional differences among three data sets (Table 4). In Table 4, if we cross-compared the age-correlated genes in three brain regions we found that BA9 has more overlap with BA47 and BA10, while BA47 and BA10 are less similar. Thus, we have a perplexing situation where we observe significant overlap between age-correlated genes among regions, but there is little overlap in significant pathways in the annotation analysis. It turns out that our network analysis on the distribution of age-correlated genes in the PPI network (Table 5) provides a possible explanation. It is well understood that degree centrality measures the amount of connecting neighborhood of a protein in the network, and therefore proteins with high degree have the potential to participate in more functional protein complexes [57]. Betweenness centrality is a “bottleneck” measure: it measures the fraction of shortest paths that pass through a given node, and thus quantifies the ability of the node to monitor communication between proteins or pathways [50]. Therefore, a node with high scores in both degree and betweenness centrality in the network could be involved in multiple protein complexes distributed across several pathways. The fact that a significant portion of age-correlated genes have this characteristic suggests that age-correlated genes in each region may function together closely or even as protein complexes, and hence different pathways were identified in each of the brain regions because they have different combinations of age-correlated genes.

We also calculated a different topological property called average clustering coefficient and found no significance in any age/region group (Table 6). One important property of the average clustering coefficient is that it measures the local “cliquishness” of a network neighborhood of a protein [58]. Mathematically, a clique is a subnetwork where every node is connected to one another; therefore the average clustering coefficient measures if all interacting partners of a gene tend to interact with each other and be part of a single complex. In our study, the z-scores of degree, betweenness, and closeness centralities of age-correlated genes were all very high, but this was not the case for the average clustering coefficient. This implies that although the numbers of interactants of age-correlated genes are high, the interactants themselves do not necessarily form complexes together; rather they participate in distinct functional pathways. This is consistent with our annotation analysis result that age-correlated genes are associated only with higher, less specific levels in Gene Ontology hierarchy.

There are certain limitations of this type of “cross-sectional” experimental design for human brain aging. First, each subject is only observed once (postmortem) and differences between individuals contribute variance to the data. Second, since we are directly measuring the physiological state, our age estimator is for the “physiological age” which will be different from the actual age of an individual. As independent age biomarkers were not available for the datasets, we use the chronological age as a “surrogate” covariate for the physiological age. Although the difference between the “physiological age” and the chronological age varies at the individual level, the difference is stochastic and will be smaller when we average over the entire population, as our analysis shows.

In addition to the intrinsic stochastic difference between physiological aging rate and actual age, there are other exogenous factors that may contribute to the age estimation errors: (1) quality and protocol for RNA extraction and profiling, and postmortem interval differ from study to study, (2) within the same study, brain region dissection may not match perfectly, (3) there may be other undiagnosed neurological conditions. For example, early-stage neurodegeneration may take place in some older subjects and perturb their expression profile substantially, yet it is hard to distinguish from the natural brain aging process, which also involves slight brain atrophy and amyloid accumulation. A larger sample size will alleviate this issue and produce more robust results.

A sensible future direction will be to perform larger studies with more brain regions and collect independent physiological age biomarkers such as MRI imaging data. Correlating brain aging with other tissue types such as blood over the same set of individuals enables us to explore common and different parts of aging rate between different tissues, but can also lead to more accessible biomarkers. A powerful approach is to integrate gene expression with genetic data to find expression quantitative trait linkage (eQTL) that influence expression of age-correlated genes; this will allow us to better pinpoint causal relations [59], [60]. We also plan to apply our naïve Bayes model to other age-informative biomarkers such as cerebrospinal fluids (CSF), plasma proteomic data, and MRI in order to better quantify the correlations between rate of aging and neurodegeneration.

## Conclusions

We have developed a naïve Bayes (NB) model for assessing the capacity of genes undergoing relatively large transitions in expression at particular age points to provide an estimate of an individual's age. When we applied our method to three brain regions located in the frontal cortex, we found a 1.46% to 34.46% improvement in chronological age estimation accuracy over other published models. We were able to obtain genes with age-correlated expression changes at particular age thresholds at a *p*-value of 0.005. We determined the genes associated with each decade by aggregating gene sets associated with age thresholds in same decade. We found that age-correlated genes are not evenly distributed on the age scale, but rather are most prominent around age 50, suggesting that important changes in basic physiology occur around this age.

## Supporting Information

Table S1 **Functional annotation analysis for three brain regions is done using NIAID DAVID online tool** **(** http://david.abcc.ncifcrf.gov/tools.jsp **).** We only listed Gene Ontology terms and KEGG pathways that have significant p-values (p-value≤0.001) and shared by at least two groups. The terms have similar function were grouped together and have enrichment score greater than 3 (See Method section).
(XLSX)

Table S2 **Topological Characteristics of the protein-protein interaction network of age-correlated genes.** Following steps in (a), up- and down- regulated age-correlated genes of each group: young, middle-age, and old.
(DOCX)

## References

[1] Masoro EJ, Austad SN (2006) Handbook of the biology of aging. Elsevier Academic Press.

[2] Nakamura E, Miyao K (2003) Further evaluation of the basic nature of the human biological aging process based on a factor analysis of age-related physiological variables. J Gerontol A Biol Sci Med Sci 58: 196–204. 12634284 10.1093/gerona/58.3.b196

[3] Ueno LM, Yamashita Y, Moritani T, Nakamura E (2003) Biomarkers of aging in women and the rate of longitudinal changes. J Physiol Anthropol Appl Human Sci 22: 37–46. 10.2114/jpa.22.37 12672981

[4] Golden TR, Hubbard A, Dando C, Herren MA, Melov S (2008) Age-related behaviors have distinct transcriptional profiles in Caenorhabditis elegans. Aging Cell 7: 850–865. 18778409 10.1111/j.1474-9726.2008.00433.x PMC2613281

[5] Cook PE, Hugo LE, Iturbe-Ormaetxe I, Williams CR, Chenoweth SF, et al (2007) Predicting the age of mosquitoes using transcriptional profiles. Nat Protoc 2: 2796–2806. 18007615 10.1038/nprot.2007.396

[6] Girardot F, Lasbleiz C, Monnier V, Tricoire H (2006) Specific age-related signatures in Drosophila body parts transcriptome. BMC Genomics 7: 69. 16584578 10.1186/1471-2164-7-69 PMC1481561

[7] Pletcher SD, Macdonald SJ, Marguerie R, Certa U, Stearns SC, et al (2002) Genome-wide transcript profiles in aging and calorically restricted Drosophila melanogaster. Curr Biol 12: 712–723. 12007414 10.1016/s0960-9822(02)00808-4

[8] Landis GN, Abdueva D, Skvortsov D, Yang J, Rabin BE, et al (2004) Similar gene expression patterns characterize aging and oxidative stress in Drosophila melanogaster. Proc Natl Acad Sci U S A 101: 7663–7668. 15136717 10.1073/pnas.0307605101 PMC419663

[9] Zhan M, Yamaza H, Sun Y, Sinclair J, Li H, et al (2007) Temporal and spatial transcriptional profiles of aging in Drosophila melanogaster. Genome Res 17: 1236–1243. 17623811 10.1101/gr.6216607 PMC1933522

[10] Fry RC, Sambandan TG, Rha C (2003) DNA damage and stress transcripts in Saccharomyces cerevisiae mutant sgs1. Mech Ageing Dev 124: 839–846. 12875747 10.1016/s0047-6374(03)00144-1

[11] Zahn JM, Poosala S, Owen AB, Ingram DK, Lustig A, et al (2007) AGEMAP: a gene expression database for aging in mice. PLoS Genet 3: e201. 18081424 10.1371/journal.pgen.0030201 PMC2098796

[12] Lu T, Pan Y, Kao SY, Li C, Kohane I, et al (2004) Gene regulation and DNA damage in the ageing human brain. Nature 429: 883–891. 15190254 10.1038/nature02661

[13] Rodwell GE, Sonu R, Zahn JM, Lund J, Wilhelmy J, et al (2004) A transcriptional profile of aging in the human kidney. PLoS Biol 2: e427. 15562319 10.1371/journal.pbio.0020427 PMC532391

[14] Zahn JM, Sonu R, Vogel H, Crane E, Mazan-Mamczarz K, et al (2006) Transcriptional profiling of aging in human muscle reveals a common aging signature. PLoS Genet 2: e115. 16789832 10.1371/journal.pgen.0020115 PMC1513263

[15] Cook PE, Hugo LE, Iturbe-Ormaetxe I, Williams CR, Chenoweth SF, et al (2006) The use of transcriptional profiles to predict adult mosquito age under field conditions. Proc Natl Acad Sci U S A 103: 18060–18065. 17110448 10.1073/pnas.0604875103 PMC1838706

[16] Erraji-Benchekroun L, Underwood MD, Arango V, Galfalvy H, Pavlidis P, et al (2005) Molecular aging in human prefrontal cortex is selective and continuous throughout adult life. Biol Psychiatry 57: 549–558. 15737671 10.1016/j.biopsych.2004.10.034

[17] Fu C, Hickey M, Morrison M, McCarter R, Han ES (2006) Tissue specific and non-specific changes in gene expression by aging and by early stage CR. Mech Ageing Dev 127: 905–916. 17092546 10.1016/j.mad.2006.09.006 PMC1764499

[18] Blalock EM, Geddes JW, Chen KC, Porter NM, Markesbery WR, et al (2004) Incipient Alzheimer's disease: microarray correlation analyses reveal major transcriptional and tumor suppressor responses. Proc Natl Acad Sci U S A 101: 2173–2178. 14769913 10.1073/pnas.0308512100 PMC357071

[19] Hawse JR, Hejtmancik JF, Horwitz J, Kantorow M (2004) Identification and functional clustering of global gene expression differences between age-related cataract and clear human lenses and aged human lenses. Exp Eye Res 79: 935–940. 15642332 10.1016/j.exer.2004.04.007 PMC1351355

[20] Golden TR, Hubbard A, Melov S (2006) Microarray analysis of variation in individual aging C. elegans: approaches and challenges. Exp Gerontol 41: 1040–1045. 16876364 10.1016/j.exger.2006.06.034

[21] Cao K, Plotkin JB, Wang LS (2010) Age-Correlated Gene Expression in Normal and Neurodegenerative Human Brain Tissues. PLoS ONE 5. 10.1371/journal.pone.0013098 PMC2947518 20927326

[22] Ho JW, Stefani M, dos Remedios CG, Charleston MA (2009) A model selection approach to discover age-dependent gene expression patterns using quantile regression models. BMC Genomics 10 Suppl 3: S16. 10.1186/1471-2164-10-S3-S16 PMC2788368 19958479

[23] Bergman A, Atzmon G, Ye K, MacCarthy T, Barzilai N (2007) Buffering mechanisms in aging: a systems approach toward uncovering the genetic component of aging. PLoS Comput Biol 3: e170. 17784782 10.1371/journal.pcbi.0030170 PMC1963511

[24] Wu JZ, Irizarry R, MacDonald J, Gentry J (2007) gcrma: Background Adjustment Using Sequence Information. R package version 2.14.

[25] Gentleman RC, Carey VJ, Bates DM, Bolstad B, Dettling M, et al (2004) Bioconductor: open software development for computational biology and bioinformatics. Genome Biol 5: R80. 15461798 10.1186/gb-2004-5-10-r80 PMC545600

[26] Friedman NG, Goldszmidt M (1997) Bayesian network classifiers. Machine Learning 29. pp. 131–163.

[27] Langley P, Iba W, Thompson K (1992) An analysis of Bayesian classifiers.

[28] Hall M (2007) A decision tree-based attribute weighting filter for naive Bayes. Knowledge-Based Systems 20: 120–126.

[29] Sandberg R, Winberg G, Branden CI, Kaske A, Ernberg I, et al (2001) Capturing whole-genome characteristics in short sequences using a naive Bayesian classifier. Genome Research 11: 1404–1409. 11483581 10.1101/gr.186401 PMC311094

[30] Efron B, Hastie T, Johnstone I, Tibshirani R (2004) Least angle regression. Annals of Statistics 32: 407–451.

[31] Kooperberg C, Bose S, Stone CJ (1997) Polychotomous regression. Journal of the American Statistical Association 92: 117–127.

[32] Keshava Prasad TS, Goel R, Kandasamy K, Keerthikumar S, Kumar S, et al (2009) Human Protein Reference Database–2009 update. Nucleic Acids Res 37: D767–772. 18988627 10.1093/nar/gkn892 PMC2686490

[33] Ashburner M, Ball CA, Blake JA, Botstein D, Butler H, et al (2000) Gene ontology: tool for the unification of biology. The Gene Ontology Consortium. Nat Genet 25: 25–29. 10802651 10.1038/75556 PMC3037419

[34] Kanehisa M, Goto S, Furumichi M, Tanabe M, Hirakawa M (2010) KEGG for representation and analysis of molecular networks involving diseases and drugs. Nucleic Acids Res 38: D355–360. 19880382 10.1093/nar/gkp896 PMC2808910

[35] Kanehisa M, Goto S, Hattori M, Aoki-Kinoshita KF, Itoh M, et al (2006) From genomics to chemical genomics: new developments in KEGG. Nucleic Acids Res 34: D354–357. 16381885 10.1093/nar/gkj102 PMC1347464

[36] Kanehisa M, Goto S (2000) KEGG: kyoto encyclopedia of genes and genomes. Nucleic Acids Res 28: 27–30. 10592173 10.1093/nar/28.1.27 PMC102409

[37] Huang da W, Sherman BT, Tan Q, Collins JR, Alvord WG, et al (2007) The DAVID Gene Functional Classification Tool: a novel biological module-centric algorithm to functionally analyze large gene lists. Genome Biol 8: R183. 17784955 10.1186/gb-2007-8-9-r183 PMC2375021

[38] Loerch PM, Lu T, Dakin KA, Vann JM, Isaacs A, et al (2008) Evolution of the aging brain transcriptome and synaptic regulation. PLoS ONE 3: e3329. 18830410 10.1371/journal.pone.0003329 PMC2553198

[39] Yankner BA, Lu T, Loerch P (2008) The aging brain. Annu Rev Pathol 3: 41–66. 18039130 10.1146/annurev.pathmechdis.2.010506.092044

[40] Reddy PH, Beal MF (2008) Amyloid beta, mitochondrial dysfunction and synaptic damage: implications for cognitive decline in aging and Alzheimer's disease. Trends Mol Med 14: 45–53. 18218341 10.1016/j.molmed.2007.12.002 PMC3107703

[41] Uauy R, Dangour AD (2006) Nutrition in brain development and aging: role of essential fatty acids. Nutr Rev 64: S24–33; discussion S72–91. 16770950 10.1301/nr.2006.may.s24-s33

[42] Bourre JM (2004) Roles of unsaturated fatty acids (especially omega-3 fatty acids) in the brain at various ages and during ageing. J Nutr Health Aging 8: 163–174. 15129302

[43] Bezard J, Blond JP, Bernard A, Clouet P (1994) The metabolism and availability of essential fatty acids in animal and human tissues. Reprod Nutr Dev 34: 539–568. 7840871 10.1051/rnd:19940603

[44] Yehuda S, Rabinovitz S, Carasso RL, Mostofsky DI (2002) The role of polyunsaturated fatty acids in restoring the aging neuronal membrane. Neurobiol Aging 23: 843–853. 12392789 10.1016/s0197-4580(02)00074-x

[45] Barrientos A, Casademont J, Cardellach F, Estivill X, Urbano-Marquez A, et al (1997) Reduced steady-state levels of mitochondrial RNA and increased mitochondrial DNA amount in human brain with aging. Brain Res Mol Brain Res 52: 284–289. 9495550 10.1016/s0169-328x(97)00278-7

[46] Shigenaga MK, Hagen TM, Ames BN (1994) Oxidative Damage and Mitochondrial Decay in Aging. Proceedings of the National Academy of Sciences of the United States of America 91: 10771–10778. 7971961 10.1073/pnas.91.23.10771 PMC45108

[47] Mecocci P, MacGarvey U, Kaufman AE, Koontz D, Shoffner JM, et al (1993) Oxidative damage to mitochondrial DNA shows marked age-dependent increases in human brain. Ann Neurol 34: 609–616. 8215249 10.1002/ana.410340416

[48] Lipinski MM, Zheng B, Lu T, Yan Z, Py BF, et al (2010) Genome-wide analysis reveals mechanisms modulating autophagy in normal brain aging and in Alzheimer's disease. Proc Natl Acad Sci U S A 107: 14164–14169. 20660724 10.1073/pnas.1009485107 PMC2922576

[49] Simpson JE, Ince PG, Shaw PJ, Heath PR, Raman R, et al (2011) Microarray analysis of the astrocyte transcriptome in the aging brain: relationship to Alzheimer's pathology and APOE genotype. Neurobiol Aging 10.1016/j.neurobiolaging.2011.04.013 21705112

[50] Yu H, Kim PM, Sprecher E, Trifonov V, Gerstein M (2007) The importance of bottlenecks in protein networks: correlation with gene essentiality and expression dynamics. PLoS Comput Biol 3: e59. 17447836 10.1371/journal.pcbi.0030059 PMC1853125

[51] Barabasi AL, Oltvai ZN (2004) Network biology: understanding the cell's functional organization. Nat Rev Genet 5: 101–113. 14735121 10.1038/nrg1272

[52] David-Eden H, Mandel-Gutfreund Y (2008) Revealing unique properties of the ribosome using a network based analysis. Nucleic Acids Res 36: 4641–4652. 18625614 10.1093/nar/gkn433 PMC2504294

[53] Tsugita A, Kawakami T, Uchida T, Sakai T, Kamo M, et al (2000) Proteome analysis of mouse brain: two-dimensional electrophoresis profiles of tissue proteins during the course of aging. Electrophoresis 21: 1853–1871. 10870971 10.1002/(SICI)1522-2683(20000501)21:9<1853::AID-ELPS1853>3.0.CO;2-Y

[54] Forester BP, Berlow YA, Harper DG, Jensen JE, Lange N, et al Age-related changes in brain energetics and phospholipid metabolism. NMR Biomed 23: 242–250. 19908224 10.1002/nbm.1444

[55] Rhein V, Song X, Wiesner A, Ittner LM, Baysang G, et al (2009) Amyloid-beta and tau synergistically impair the oxidative phosphorylation system in triple transgenic Alzheimer's disease mice. Proc Natl Acad Sci U S A 106: 20057–20062. 19897719 10.1073/pnas.0905529106 PMC2774257

[56] Wang X, Michaelis EK (2010) Selective neuronal vulnerability to oxidative stress in the brain. Front Aging Neurosci 2: 12. 20552050 10.3389/fnagi.2010.00012 PMC2874397

[57] Manke T, Demetrius L, Vingron M (2005) Lethality and entropy of protein interaction networks. Genome Inform 16: 159–163. 16362918

[58] Spirin V, Mirny LA (2003) Protein complexes and functional modules in molecular networks. Proc Natl Acad Sci U S A 100: 12123–12128. 14517352 10.1073/pnas.2032324100 PMC218723

[59] Cookson W, Liang L, Abecasis G, Moffatt M, Lathrop M (2009) Mapping complex disease traits with global gene expression. Nat Rev Genet 10: 184–194. 19223927 10.1038/nrg2537 PMC4550035

[60] Pickrell JK, Marioni JC, Pai AA, Degner JF, Engelhardt BE, et al (2010) Understanding mechanisms underlying human gene expression variation with RNA sequencing. Nature 464: 768–772. 20220758 10.1038/nature08872 PMC3089435

