# Supplementary material for: Analysis of Nonlinear Gene Expression Progression Reveals Extensive Pathway and Age-Specific Transitions in Aging Human Brains
Source: PLoS One. 2013 Oct 3;8(10):e74578. doi: 10.1371/journal.pone.0074578 (PMC3789733; doi:10.1371/journal.pone.0074578)
Supplement: Table S2 — Topological Characteristics of the protein-protein interaction network of age-correlated genes. Following steps in (a), up- and down- regulated age-correlated genes of each group: young, middle-age, and old. (DOCX) [file pone.0074578.s002.docx]

**Table S2.** Topological Characteristics of the protein-protein interaction network of age-correlated genes. Following steps in (a), up- and down- regulated age-correlated genes of each group: young, middle-age, and old.

|  | Topological Characteristics in Protein-Protein Interaction Network | | | | | | | | | | | | |
| --- | --- | --- | --- | --- | --- | --- | --- | --- | --- | --- | --- | --- | --- |
|  | Degree | | | Betweenness Centrality | | | Closeness Centrality | | | | Clustering Coefficient | | |
|  | Young | Middle-aged | Old | Young | Middle-aged | Old | Young | Middle-aged | Old | | Young | Middle-aged | Old |
| BA10 dn | 19.475 | 19.750 | 19.797 | 1.08E-3 | 1.11E-3 | 1.10E-3 | 0.262 | 0.263 | 0.263 | | 0.129 | 0.128 | 0.134 |
| Z-score | 17.44 | 18.67 | 16.65 | 11.31 | 12.33 | 10.80 | 15.67 | 17.17 | 14.16 | | 2.44 | 2.57 | 2.78 |
| BA10 up | 18.636 | 19.863 | 19.314 | 9.85E-4 | 1.09E-3 | 1.08E-3 | 0.261 | 0.263 | | 0.262 | 0.132 | 0.125 | 0.136 |
| Z-score | 13.91 | 20.58 | 15.22 | 8.31 | 12.79 | 9.72 | 13.15 | 17.89 | | 13.60 | 2.51 | 2.32 | 2.90 |
|  |  |  |  |  |  |  |  |  | |  |  |  |  |
| BA9 dn | 14.667 | 13.043 | 13.455 | 8.36E-4 | 7.05E-4 | 7.35E-4 | 0.256 | 0.256 | | 0.256 | 0.076 | 0.076 | 0.076 |
| *Z*-score | 6.64 | 7.51 | 7.46 | 4.80 | 5.31 | 5.31 | 7.31 | 10.06 | | 10.27 | -1.97 | -2.95 | -2.91 |
| BA9 up | 15.304 | 13.241 | 13.094 | 9.23E-4 | 7.21E-4 | 7.18E-4 | 0.258 | 0.255 | | 0.255 | 0.077 | 0.077 | 0.079 |
| Z-score | 7.11 | 7.76 | 6.28 | 5.65 | 5.50 | 4.47 | 8.21 | 10.25 | | 8.63 | -1.88 | -2.95 | -2.46 |
|  |  |  |  |  |  |  |  |  | |  |  |  |  |
| BA47 dn | 11.560 | 10.732 | 7.568 | 6.88E-4 | 5.46E-4 | 3.14E-4 | 0.247 | 0.247 | | 0.243 | 0.114 | 0.100 | 0.084 |
| Z -score | 4.58 | 5.42 | -0.55 | 4.47 | 3.96 | -0.47 | 3.94 | 6.06 | | 1.27 | 0.789 | -0.70 | -1.93 |
| BA47 up | 10.760 | 10.232 | 10.433 | 5.39E-4 | 5.05E-4 | 4.95E-4 | 0.246 | 0.246 | | 0.247 | 0.08 | 0.104 | 0.094 |
| Z-score | 3.00 | 3.39 | 2.27 | 1.99 | 2.34 | 1.31 | 3.02 | 3.57 | | 2.65 | -1.58 | -0.14 | -0.76 |
